# Association of prolactin receptor (*PRLR*) variants with prolactinomas

**DOI:** 10.1093/hmg/ddy396

**Published:** 2018-11-15

**Authors:** Caroline M Gorvin, Paul J Newey, Angela Rogers, Victoria Stokes, Matt J Neville, Kate E Lines, Georgia Ntali, Peter Lees, Patrick J Morrison, Panagiotis N Singhellakis, Fotini Ch Malandrinou, Niki Karavitaki, Ashley B Grossman, Fredrik Karpe, Rajesh V Thakker

**Affiliations:** 1 Academic Endocrine Unit, Oxford Centre for Diabetes, Endocrinology and Metabolism, Radcliffe Department of Medicine, University of Oxford, Oxford, UK; 2 Oxford NIHR Biomedical Research Centre, University of Oxford, Churchill Hospital, Oxford, UK; 3 Metabolic Research Group, Oxford Centre for Diabetes, Endocrinology and Metabolism, Radcliffe Department of Medicine, University of Oxford, Oxford, UK; 4 Department of Neurosurgery, Southampton General Hospital, Southampton, Hampshire; 5 Northern Ireland Regional Genetics Centre, Belfast City Hospital, Lisburn Road, Belfast, UK; 6 Department of Endocrinology, Metabolism and Diabetes Mellitus, St Savvas Cancer Hospital, Athens, Greece; 7 Department of Endocrinology, Oxford Centre for Diabetes, Endocrinology and Metabolism, Churchill Hospital, Oxford, UK

## Abstract

Prolactinomas are the most frequent type of pituitary tumors, which represent 10–20% of all intracranial neoplasms in humans. Prolactinomas develop in mice lacking the prolactin receptor (PRLR), which is a member of the cytokine receptor superfamily that signals via Janus kinase-2-signal transducer and activator of transcription-5 (JAK2-STAT5) or phosphoinositide 3-kinase-Akt (PI3K-Akt) pathways to mediate changes in transcription, differentiation and proliferation. To elucidate the role of the *PRLR* gene in human prolactinomas, we determined the *PRLR* sequence in 50 DNA samples (35 leucocytes, 15 tumors) from 46 prolactinoma patients (59% males, 41% females). This identified six germline *PRLR* variants, which comprised four rare variants (Gly57Ser, Glu376Gln, Arg453Trp and Asn492Ile) and two low-frequency variants (Ile76Val, Ile146Leu), but no somatic variants. The rare variants, Glu376Gln and Asn492Ile, which were in complete linkage disequilibrium, and are located in the PRLR intracellular domain, occurred with significantly higher frequencies (*P* < 0.0001) in prolactinoma patients than in 60 706 individuals of the Exome Aggregation Consortium cohort and 7045 individuals of the Oxford Biobank. *In vitro* analysis of the PRLR variants demonstrated that the Asn492Ile variant, but not Glu376Gln, when compared to wild-type (WT) PRLR, increased prolactin-induced pAkt signaling (>1.3-fold, *P* < 0.02) and proliferation (1.4-fold, *P* < 0.02), but did not affect pSTAT5 signaling. Treatment of cells with an Akt1/2 inhibitor or everolimus, which acts on the Akt pathway, reduced Asn492Ile signaling and proliferation to WT levels. Thus, our results identify an association between a gain-of-function PRLR variant and prolactinomas and reveal a new etiology and potential therapeutic approach for these neoplasms.

## Introduction

Prolactinomas account for ~40% of all pituitary tumors that represent 10–20% of all intracranial neoplasms in humans and as such are the third most common type of primary brain tumors after gliomas and meningiomas (1–3). Prolactinomas hypersecrete the hormone prolactin, and the resulting elevated serum prolactin concentrations in patients may be associated in women with menstrual irregularities, infertility and galactorrhea and in men with reduced libido or erectile dysfunction (3,4). Prolactinomas vary in size, which is usually assessed by magnetic resonance imaging, and those <10 mm or >10 mm in diameter are referred to as microprolactinomas or macroprolactinomas, respectively (3,4). Macroprolactinomas in both genders may compress the adjacent optic chiasma and cause a visual field defect (3,4). Prolactinomas in ~5% of patients may occur as a hereditary disorder and be due to germline mutations of the multiple endocrine neoplasia type-1 (*MEN1*) or aryl hydrocarbon receptor interacting protein (*AIP*) genes (4).

Prolactin, which is secreted by lactotroph cells of the anterior pituitary and is required for induction and maintenance of lactation in the peripartum and postpartum periods (5), binds the prolactin receptor (PRLR). The PRLR, which functions as a dimer, is a class I cytokine receptor that has a multi-domain structure consisting of a ligand-binding extracellular domain (ECD, residues 1–210), a single transmembrane segment (residues 211–234) and an intracellular domain (ICD, residues 235–598) (Supplementary Material, Fig. S1). The ECD comprises two subdomains designated D1 (residues 1–101) and D2 (residues 109–210) (6–8), which are important in ligand binding and subsequent PRLR activation (6–10), and the ICD is involved in activation of signaling pathways that include the JAK2-STAT5 pathway, as well as the PI3K/Akt and MAPK pathways (Supplementary Material, Fig. S1) (9,11,12). These signaling pathways lead to transcription of target genes that regulate proliferation, differentiation and cell survival (9,11,12).

PRLRs are widely expressed in organs and tissues that include the mammary gland, reproductive system, central nervous system [e.g. the tuberoinfundibular dopamine (TIDA) neurons], pituitary, adrenal cortex, skin, bone, lung, heart, liver, pancreas, gastro-intestinal tract, lymph glands and spermatozoa (13). The precise roles of PRLRs in these tissues remain to be defined. However, PRLRs are involved in a negative feedback mechanism that regulates prolactin secretion, which is tonically inhibited by dopamine released from TIDA neurons of the hypothalamic arcuate nucleus that act upon dopamine D2 receptors on the pituitary gland (14). Thus, prolactin binds to PRLRs on the TIDA neurons that results in an increase in dopamine secretion, which reduces prolactin secretion by the pituitary. Support for this mechanism is provided by studies of mice lacking the dopamine D2 receptor, which are hyperprolactinaemic due to loss of dopaminergic inhibition at the pituitary gland (15). Interestingly, PRLR has also been reported to be expressed on lactotrophs of the pituitary gland where it may provide an autocrine loop to regulate lactotroph function (16). Moreover, human and rodent studies have reported roles for the PRLR in reproduction and development of breast and prostate tumors (17–19). For example, two low-frequency [defined as having a minor allele frequency (MAF) of 1–5%] PRLR variants, Ile76Val and Ile146Leu, have been reported to result in a gain of function with constitutive activity and to occur in 15% of a cohort of French women with multiple fibroadenomas of the breast (OMIM #615554) (17,20). However, other studies in North American, German and Polish women have not detected such associations (21–24). In addition, a loss-of-function *PRLR* mutation His188Arg mutation (H188R), which is located in the ECD and abolishes JAK2/STAT5 signaling, has been reported to occur in one family with autosomal dominant hyperprolactinemia (OMIM #615555) and to be associated with oligomenorrhea and infertility (25). The findings in this family with autosomal dominant hyperprolactinemia are consistent with those from studies of mutant mouse models that were deleted for *Prlr* alleles (18). Thus, *Prlr^+/−^* and *Prlr^−/−^* female mice have been reported to have impaired mammary gland development, while *Prlr^−/−^* female and male mice also developed hyperprolactinemia, with pituitary hyperplasia and tumors, and infertility (18). Furthermore, neuron-specific conditional *Prlr* knockout mice have also been reported to develop hyperprolactinemia and abnormalities of the estrous cycle (26), with lactotroph-specific conditional *Prlr* knockout mice having normal circulating prolactin levels and estrous cycles, but impaired dopaminergic tone (16). Prolactin has been reported to have proapoptotic and antiproliferative effects in rats (27), and investigations of *Prlr^−/−^* mice have indicated that it is the chronic downregulation of PRLR signaling in these pathways that may cause pituitary hyperplasia and prolactinoma development (28). We therefore investigated the hypothesis that *PRLR* variants, resulting in aberrant PRLR signaling, may be associated with prolactinoma and hyperprolactinaemia in humans. Surprisingly, a previous French study has reported an absence of an association between PRLR variants and prolactinomas in humans (29). However, here we show that germline Glu376Gln and Asn492Ile PRLR ICD variants, which are rare variants (defined as having a MAF <1%) and in complete linkage disequilibrium (LD), are significantly associated with occurrence of prolactinomas in humans. In addition, we show that the Asn492Ile PRLR variant is associated with increased signaling by the Akt pathway and that everolimus, a mammalian target of rapamycin (mTOR) inhibitor, is effective in normalizing this gain of Akt activity.

**Figure 1:**
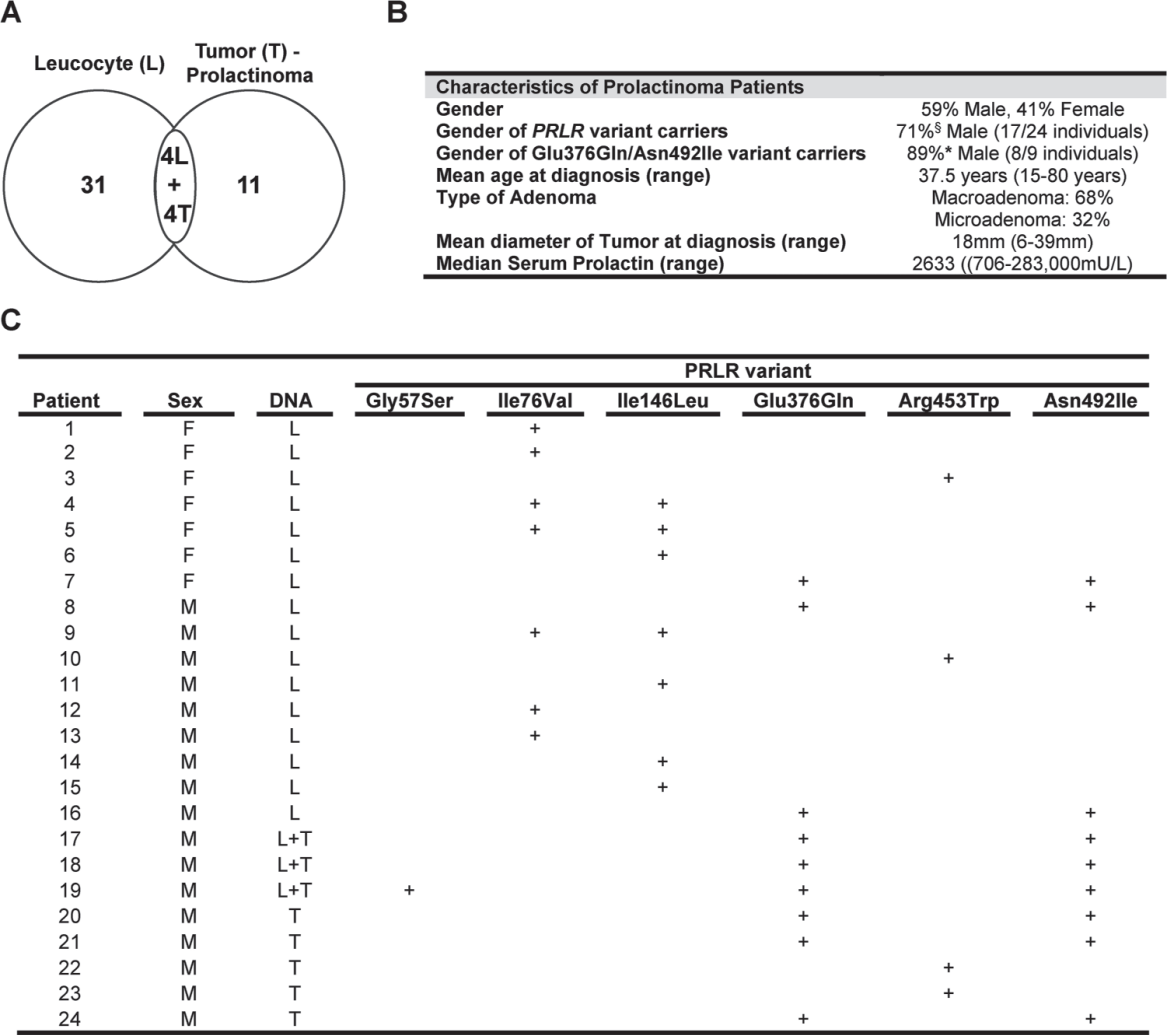
Clinical features of patients with prolactinomas. (**A**) Distribution of 50 DNA samples (35 leucocytes (L) and 15 tumors (T)) from 46 patients with prolactinomas. Matched leucocyte and tumor (L + T) samples were obtained from four unrelated patients. (**B**) Clinical details of the prolactinoma cohort. No patients had MEN1. Four patients had a history of familial prolactinoma, with two patients being the father and daughter from one family, both of whom had none of the rare (<1%) or low-frequency (<5%) PRLR variants, and the other two patients being first cousins from another family, both of whom had the low-frequency PRLR variant Ile76Val. All patients had hyperprolactinemia (prolactin normal ranges: 45–375 mU/L (2.1–17.7 ng/mL) for males and 60–625 mU/L (2.8–29.5 ng/mL) for females; for conversion to ng/mL, divide by 21.2). (**C**) Details of patients with prolactinomas in whom rare or low-frequency PRLR variants were identified. The rare PRLR variants are Gly57Ser, Glu376Gln, Arg453Trp and Asn492Ile, while the Ile76Val and Ile146Leu represent low-frequency variants. The Glu376Gln and Asn492Ile variants, which are in perfect LD, were observed in the same nine individuals. Combined analysis of leucocyte and prolactinoma tumor DNA from four patients revealed the presence of identical genotypes and the absence of somatic mutations in the tumor. F, female; M, male. + indicates presence of common and low-frequency/rare variants at each codon.

**Figure 2:**
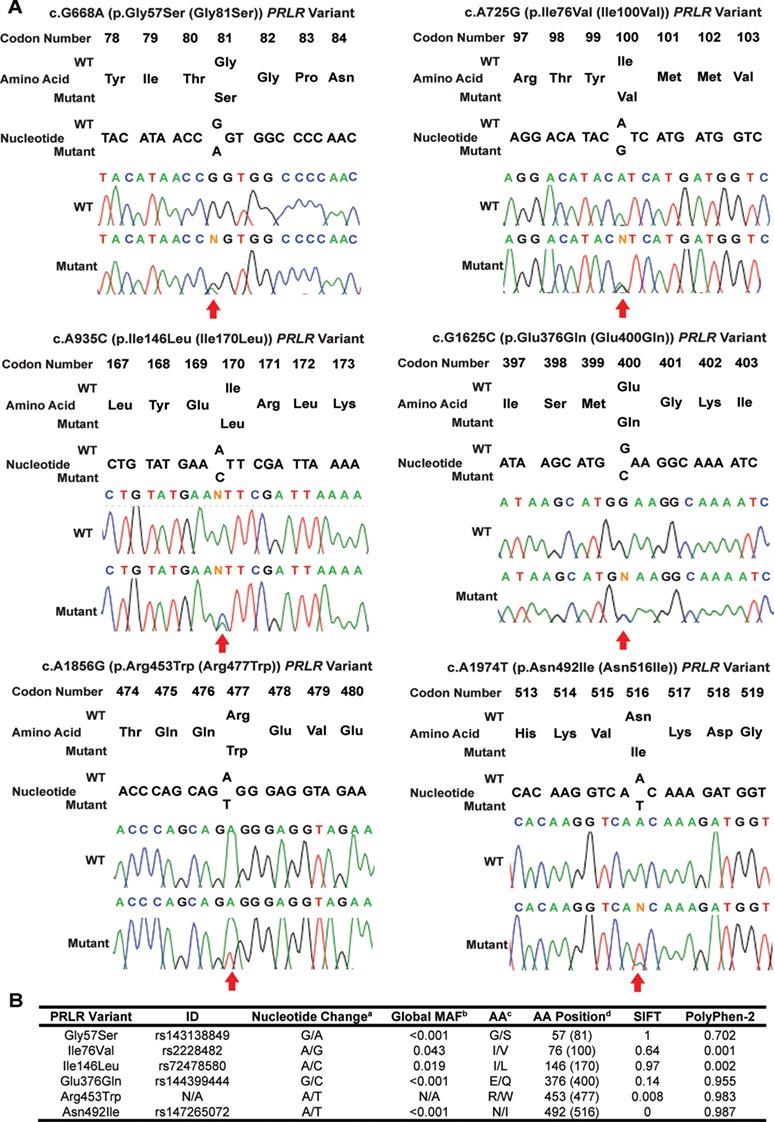
Identification of *PRLR* variants in prolactinoma patients. (**A**) DNA sequence analysis in 46 prolactinoma patients identified six *PRLR* variants. These included three ECD variants: Gly57Ser (upper left panel), Ile76Val (upper right panel) and Ile146Leu (middle left panel); and three ICD variants: Glu376Gln (middle right panel), Arg453Trp (lower left panel) and Asn492Ile (lower right panel). Amino acid and nucleotide numbering is shown above chromatograms. For each chromatogram, codon numbers include the 24 amino acid signal peptide. The Arg453Trp variant has not previously been reported in online databases. Each chromatogram is from leucocyte DNA. (**B**) Global MAF and pathogenicity prediction of the six *PRLR* variants. ^a^Reference allele/minor allele; ^b^MAF of all ethnic groups combined; ^c^Amino acid represented by reference allele/amino acid represented by minor allele; and ^d^Amino acid number in mature protein without the signal peptide, and with the amino acid number according to the full-length 622 amino acid PRLR protein (NCBI, NP_000940; UniProt P16471) in parentheses. Rare, low-frequency and common variants are defined to have a MAF of <1%, 1–5% and >5%, respectively (30). SIFT and Polyphen-2 scores predict the effect of amino acid substitutions (52,53). SIFT predicts effects based on sequence homology and physicochemical properties and gives a qualitative (either tolerated or deleterious) and quantitative score (probability that the change is tolerated, i.e. the nearer to 0 the more likely deleterious, and the nearer to 1 the more likely benign) (53). Polyphen-2 predicts effects based on sequence homology, protein databank structures and protein family annotations (52). Polyphen-2 gives a qualitative score of probably damaging, possibly damaging, benign or unknown, and quantitative scores are based on the probability that the change is damaging, i.e. the nearer to 0 the more benign. A mutation is classified as probably damaging if the score is >0.85, and possibly damaging if the score is >0.15 (52).

**Table 1:** Frequency of non-synonymous missense PRLR variants in prolactinoma patients

**Prolactinoma patients**										**ExAc** ^**a**^	**OBB** ^**b**^
**PRLR Variant**	**L + T (n = 46 patients)** ^**c**^			**L (n = 35 samples)** ^**d**^			**T (n = 15 samples)** ^**e**^			***n* = 60 706**	***n* = 7045**
	**Number (MAF)** ^**f**^	**Fisher’s exact test**		**Number (MAF)**	**Fisher’s exact test**		**Number (MAF)**	**Fisher’s exact test**		**Number (MAF)**	**Number (MAF)**
		**ExAc**	**OBB**		**ExAc**	**OBB**		**ExAc**	**OBB**		
**Gly57Ser**	1 (0.01)	NS	NS	1 (0.02)	NS	NS	1 (0.04)	0.022	0.025	14 (0.0001)	1 (0.00005)
**Ile76Val**	7 (0.08)	NS	NS	7 (0.10)	NS	0.02	0 (0)	NS	NS	5274 (0.04)	411 (0.03)
**Ile146Leu**	7 (0.08)	NS	0.01	7 (0.10)	0.0018	NS	0 (0)	NS	NS	2305 (0.02)	412 (0.03)
**Glu376Gln**	9 (0.10)	<0.0001	<0.0001	6 (0.09)	<0.0001	<0.0001	6 (0.2)	<0.0001	<0.0001	108 (0.0009)	28 (0.002)
**Arg453Trp**	4 (0.04)	<0.0001	N/A	2 (0.03)	<0.0001	N/A	2 (0.07)	<0.0001	N/A	0 (0)	- (-)
**Asn492Ile**	9 (0.10)	<0.0001	<0.0001	6 (0.09)	<0.0001	<0.0001	6 (0.2)	<0.0001	<0.0001	106 (0.0009)	28 (0.002)

a ^a^ExAc contains DNA sequence data from 60 706 individuals, without known pituitary tumors, and thus provides a useful reference set of control allele frequencies for this study. ^b^OBB cohort (31) consists of an age-stratified random sample of 7045 healthy men and non-pregnant women (aged 30–50 years of European origin), thereby representing a population of similar ethnicity to prolactinoma patients within this study. Statistical analyses were performed by Fisher’s exact test. ^c^In the prolactinoma cohort of 46 patients, the occurrences of the PRLR ICD rare variants Glu376Gln and Asn492Ile were significantly greater than in the ExAc and OBB cohorts (Fig 1); and the PRLR ICD rare variant Arg453Trp, which was not available (N/A) on the OBB exome chip, was also significantly greater than in the ExAc cohort; while the PRLR ECD low-frequency variant Ile146Leu had a greater occurrence only when compared to the OBB cohort. The samples from the 46 patients consisted of leucocyte (L) DNA samples from 35 patients and 11 tumor (T) (prolactinoma) samples from unrelated patients. The associations between prolactinomas and the PRLR ICD variants remained significant in subanalyses of ^d^35 leucocyte DNA samples from 31 patients from whom only leucocyte DNA was available, and 4 patients from whom both leucocyte and tumor DNA were available; and ^e^15 tumor (prolactinoma) DNA samples from unrelated patients (representing 11 patients for whom only tumor DNA was available, and 4 patients for whom both leucocyte and tumor DNA were available). ^f^MAF for rare, low-frequency and common variants is defined as <1%, 1–5% and >5%, respectively (30). NS, not significant.

**Figure 3:**
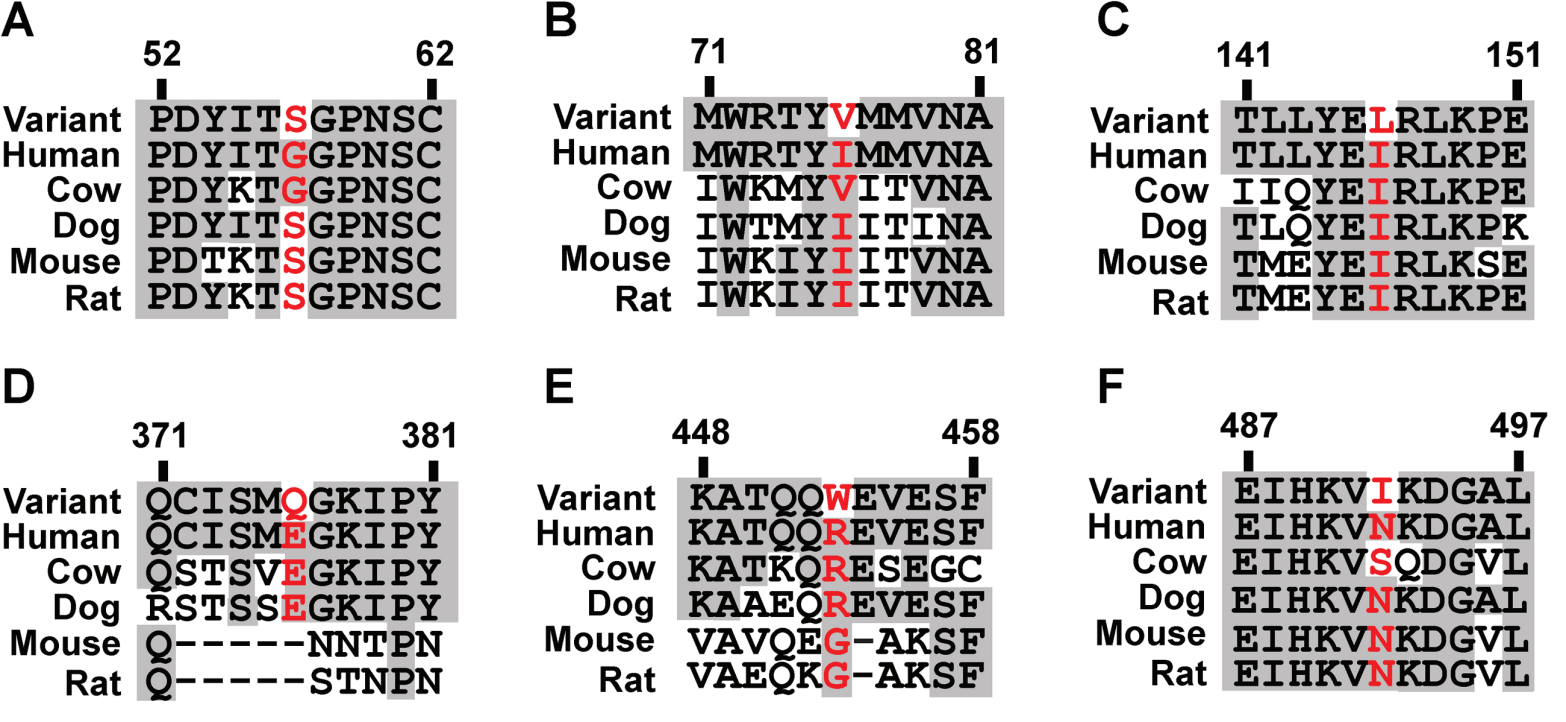
Multiple sequence alignment of PRLR protein sequence in five mammalian species, using ClustalW2 for the six PRLR variants identified in the prolactinoma patients. (**A**) Gly57Ser (G57S). (**B**) Ile76Val (I76V). (**C**) Ile146Leu (I146L). (**D**) Glu376Gln (E376Q). (**E**) Arg453Trp (R453W). (**F**) Asn492Ile (N492I). Conserved residues are shown in gray, and WT and mutant residues are shown in red.

## Results

### PRLR gene in prolactinoma patients

We determined the sequence of the *PRLR* gene in 50 DNA samples (35 leucocytes and 15 tumors) obtained from 46 patients (59% males, 41% females, average age of diagnosis = 37.5 years) with prolactinomas in whom mutations of the *MEN1* and *AIP* genes had been excluded (Fig. 1). Four of these patients had a history of familial prolactinoma, with two patients being the father and daughter from one family and the other two patients being first cousins from another family. This identified the presence of six germline *PRLR* coding variants, which comprised four rare variants (Gly57Ser, Glu376Gln, Arg453Trp and Asn492Ile) with a MAF of <1% and two low-frequency variants (Ile76Val and Ile146Leu) with a MAF of 1–5% (30) (Figs 1 and 2). Combined analyses of leucocyte and prolactinoma DNA from 4 patients and that of 11 tumor DNA samples did not identify any additional tumor-specific *PRLR* variants, thereby indicating that these *PRLR* variants are germline and that somatic *PRLR* mutations are unlikely to be involved in the development of prolactinomas in humans (Fig. 1). Three of the PRLR variants (Gly57Ser, Ile76Val and Ile146Leu) were located in the ECD, while the other three (Glu376Gln, Arg453Trp and Asn492Ile) were located in the ICD (Supplementary Material, Fig. S1). Each of the three PRLR ICD rare variants (Glu376Gln, Arg453Trp and Asn492Ile) were observed at significantly higher frequencies in the 46 prolactinoma patients when compared to their frequencies in the Exome Aggregation Consortium (ExAc) cohort (Fig. 1, Table 1). The Glu376Gln and Asn492Ile variants were found not to occur in the four patients with familial prolactinoma. The Arg453Trp variant, which was observed in four samples (two leucocyte and two tumors) from unrelated individuals, was absent in the ExAc database, and hence represented a novel variant. These four patients with the Arg453Trp variants did not have any other rare or low-frequency variants. In addition, the co-occurrence of the Glu376Gln and Asn492Ile in nine individuals in the prolactinoma cohort indicated that these two rare variants are in a high degree of LD. This is supported by the observation that identical numbers of individuals of European descent harboured each of the Glu376Gln and Asn492Ile variants in the ExAC population, while the variants were also observed in perfect LD (*r*^2^ = 1/D’ = 1) in the Oxford Biobank (OBB) cohort. The frequencies of the co-occurring Glu376Gln and Asn492Ile PRLR ICD rare variants were significantly higher in the prolactinoma patients than in the OBB cohort (31), thereby confirming the association between these two ICD PRLR variants and prolactinomas (Table 1). Furthermore, ~90% (8/9) of the Glu376Gln and Asn492Ile PRLR variants occurred in male prolactinoma patients (Fig. 1), and in 40% (6/15) of patients who had required pituitary surgery, indicating that these two PRLR variants may be associated with aggressive or medically non-responsive prolactinomas. These associations between prolactinomas and the two ICD PRLR rare variants (Glu376Gln and Asn492Ile) remained significant in separate sub-analyses of leucocyte and tumor DNA from 35 and 15 patients, respectively (Table 1). The three PRLR ICD rare variants (Glu376Gln, Arg453Trp and Asn492Ile) were predicted by SIFT and/or PolyPhen-2 to be damaging and to have partial evolutionary conservation (Figs 2 and 3). Weaker and variable associations between the prolactinomas and the PRLR ECD variants (Gly57Ser, Ile76Val and Ile146Leu) were also observed (Table 1). The ECD Gly57Ser rare variant was predicted, by SIFT and/or PolyPhen-2, to be damaging, while the ECD Ile76Val and Ile146Leu low-frequency variants were predicted to be benign (Fig. 2).

**Figure 4:**
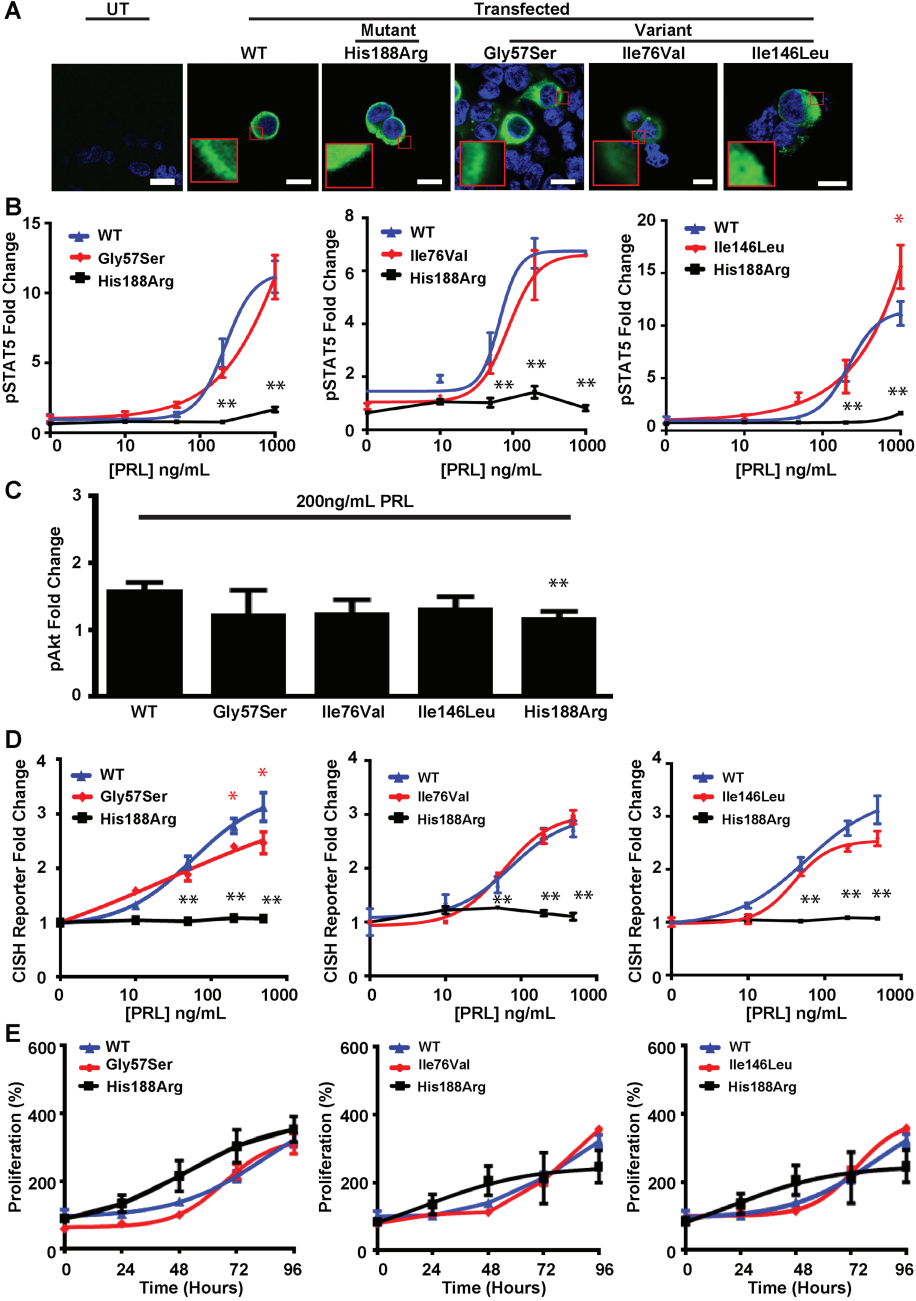
Effects of the ECD PRLR variants (Gly57Ser, Ile76Val, Ile146Leu) and mutant His188Arg PRLR on pSTAT5, pAkt and proliferation pathways. (**A**) Confocal images of PRLR (green) and DAPI (blue) in transfected HEK293 cells. Untransfected (UT) cells demonstrated no endogenous PRLR expression. Bar indicates 10 μm. Inset, zoomed image showing similar cell surface and cytoplasmic expression of PRLRs in cells transfected with WT, mutant His188Arg or variant PRLR constructs. (**B**) pSTAT5 responses following prolactin (PRL) treatment in cells expressing WT, mutant His188Arg or variant Gly57Ser (left panel), Ile76Val (middle panel) and Ile146Leu (right panel) PRLRs. PRL-induced pSTAT5 production was abolished in His188Arg and significantly increased at PRL 1000 ng/mL only in Ile146Leu cells, compared to WT. (**C**) PRL-induced AlphaScreen pAkt responses were significantly impaired in cells expressing mutant His188Arg PRLRs, but not ECD PRLR variants compared to cells expressing WT. (**D**) CISH reporter activity in cells transfected with WT, mutant His188Arg or variant Gly57Ser (left panel), Ile76Val (middle panel) or Ile146Leu (right panel) PRLRs. CISH reporter activity was significantly reduced in Gly57Ser (at PRL = 500–1000 ng/mL) and abolished in His188Arg expressing cells, compared to WT cells. (**E**) Effect of PRL (200 ng/mL) on proliferation, assessed using CellTiter Blue assays, over 96 h in cells expressing WT, mutant His188Arg or variant Gly57Ser (left panel), Ile76Leu (middle panel) and Ile146Leu (right panel) PRLRs. Proliferation was similar in variant, mutant and WT cells. Mean ± SEM from four biological replicates, with ^*^P < 0.05 and ^**^P < 0.02 for WT versus Gly57Ser, Ile76Val or Ile146Leu (red) and WT versus His188Arg cells (black). Equal expression of WT, mutant and variant PRLRs was confirmed by western blot analysis (Supplementary Material, Fig. S2).

**Figure 5:**
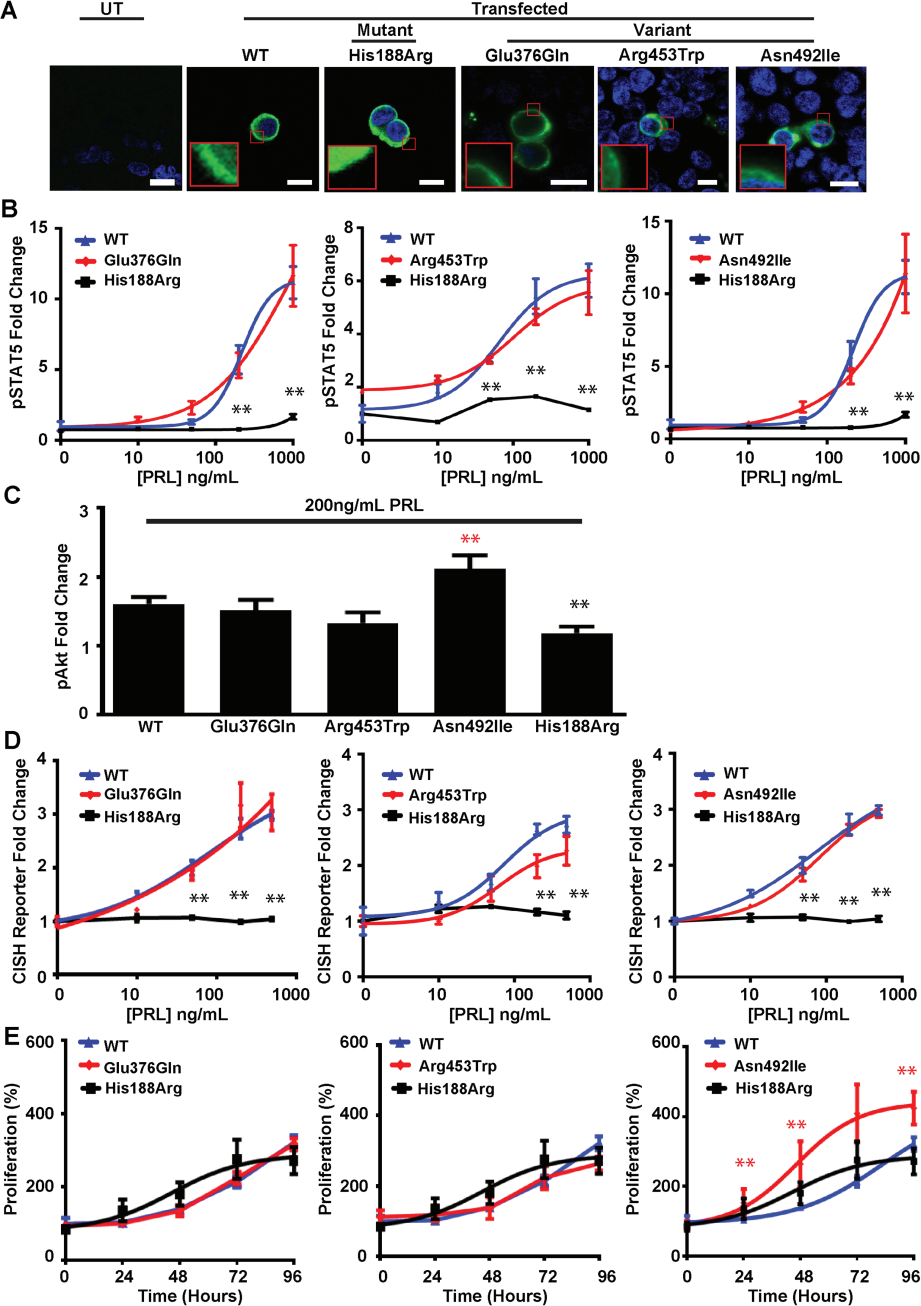
Effects of ICD PRLR variants (Glu376Gln, Arg453Trp, Asn492Ile) and ECD mutant His188Arg PRLR on pSTAT5, pAkt and proliferation pathways. (**A**) Confocal images of PRLR (green) and DAPI (blue) in transfected HEK293 cells. UT cells demonstrated no endogenous PRLR expression. Bar indicates 10 μm. Inset, zoomed image showing similar cell surface and cytoplasmic expression of PRLRs in cells transfected with WT, His188Arg, Glu376Gln, Arg453Trp or Asn492Ile PRLR constructs. (**B**) pSTAT5 responses following PRL treatment in cells expressing WT, mutant His188Arg or variant Glu376Gln (left panel), Arg453Trp (middle panel) and Asn492Ile (right panel) PRLRs. WT and ICD PRLR variants had similar PRL-induced pSTAT5 production. (**C**) PRL-induced pAkt responses in cells expressing WT, mutant His188Arg or ICD variant PRLRs. pAkt responses were significantly impaired in His188Arg cells and increased in Asn492Ile expressing cells, compared to WT cells. (**D**) CISH reporter activity in cells transfected with WT, mutant His188Arg or variant Glu376Gln (left panel), Arg453Trp (middle panel) or Asn492Ile (right panel) PRLRs. PRL-induced increased CISH reporter activity in WT, Glu376Gln, Arg453Trp or Asn492Ile cells, but not His188Arg cells. (**E**) Effect of PRL (200 ng/mL) on proliferation, assessed using CellTiter Blue assays, over 96 h in cells expressing WT, mutant His188Arg or variant Glu376Gln (left panel), Arg453Trp (middle panel) and Asn492Ile (right panel) PRLRs. Proliferation was significantly increased in Asn492Ile expressing cells compared to WT cells. Mean ± SEM from four biological replicates shown with ^*^P < 0.05 and ^**^P < 0.02 for WT versus Glu376Gln, Arg453Trp or Asn492Ile (red) and WT versus His188Arg cells (black). Equal expression of WT, mutant and variant PRLRs was confirmed by western blot analysis (Supplementary Material, Fig. S2).

### Effects of the PRLR variants on the JAK-STAT and Akt signaling pathways

**Figure 6:**
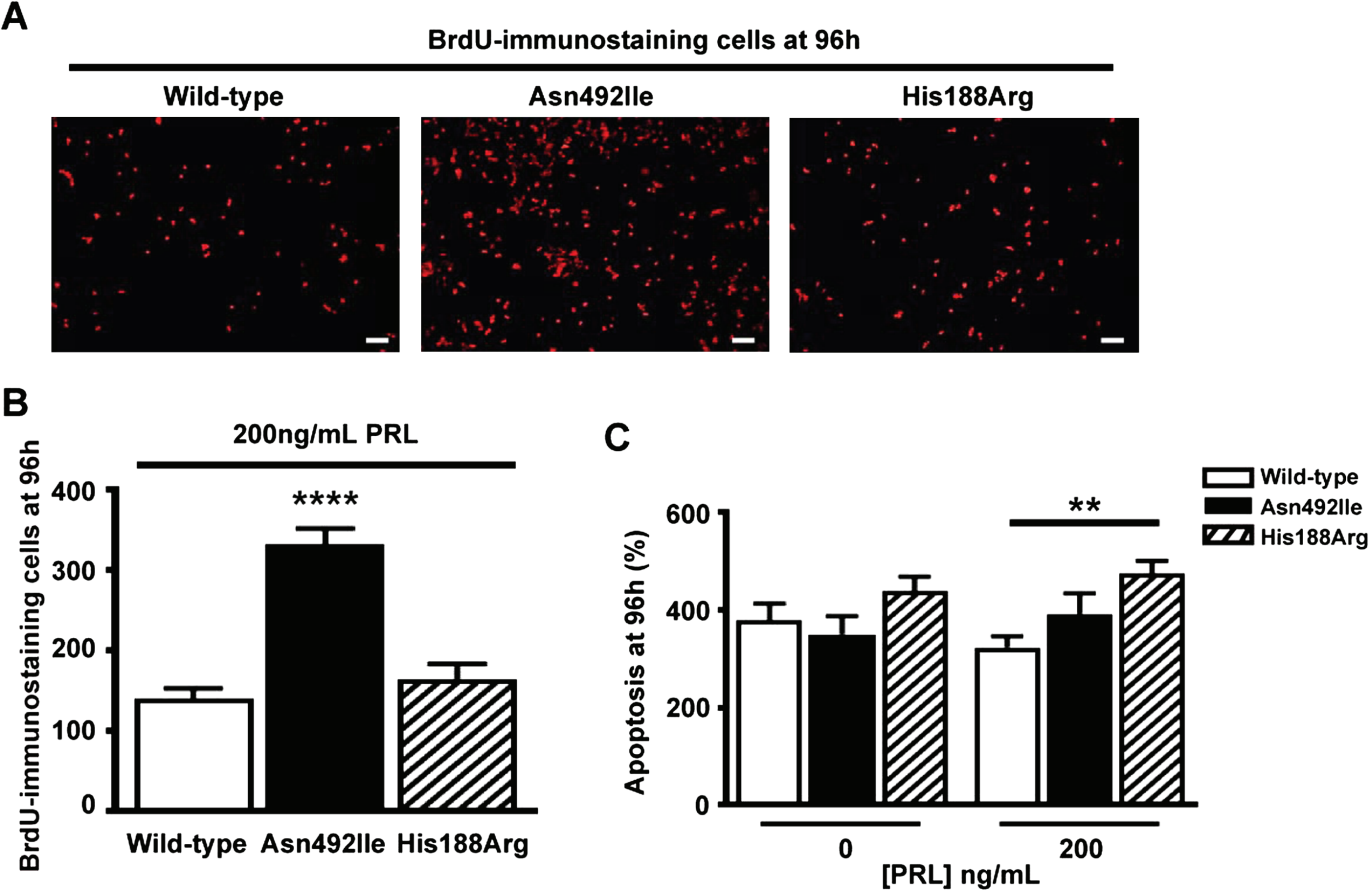
Effects of the Asn492Ile PRLR variant and His188Arg mutant on proliferation, assessed by BrdU incorporation, and on apoptosis, assessed by Caspase-3/7 assays. (**A**) Representative images showing BrdU-immunostaining in HEK293 cells that were transfected with WT, variant Asn492Ile or mutant His188Arg PRLR. Following transfection, the HEK293 cells were exposed to 200 ng/mL PRL, and at 96 h incubated with BrdU for 15 min to assess the number of proliferating cells. Bar indicates 20 μm. (**B**) Quantification of the number of BrdU-immunostained cells. Cells transfected with the Asn492Ile PRLR variant showed significantly increased BrdU immunostaining, when compared to cells transfected with WT PRLR or the mutant His188Arg PRLR, thereby confirming that the Asn492Ile PRLR variant is associated with increased proliferation (Fig. 5E). Mean ± SEM from *N* = 5 coverslips per construct, with four images taken per coverslip. Coverslips were prepared from independent transfections that were performed on two separate days. ^****^P < 0.0001. (**C**) Quantification of Caspase-3/7-mediated apoptosis activity in transfected HEK293 cells. Cells were transfected with WT, variant Asn492Ile or mutant His188Arg, treated with 0 ng/mL PRL or 200 ng/mL PRL and assessed for apoptosis at 96 h. Data were normalized to the number of proliferating cells at hour 0. Following treatment with PRL, cells expressing the His188Arg PRLR mutant had significantly more apoptosis than WT cells or cells expressing the Asn492Ile variant. Mean ± SEM for four apoptosis assays with each assay performed with four technical replicates. ^**^P < 0.01.

The PRLR can signal by STAT5 and PI3K/Akt and the effects of the six PRLR variants on these signaling pathways were therefore assessed by studying the following: the cellular expression of the PRLR; the immediate effects of prolactin binding on pSTAT5 and pAkt activation; and the later downstream effects of receptor activation on transcription of the STAT5 target gene cytokine inducible SH2-containing protein (CISH), cellular proliferation and apoptosis (Fig. 4A–E, 5A–E, 6 and Supplementary Material, Fig. S2). The effects on PRLR signaling were assessed together with that of the His188Arg mutant PRLR ECD that has been reported to result in a loss of function in association with familial hyperprolactinaemia (25). All of the PRLR variants had similar cell surface expression, cytoplasmic expression and total protein expression, when compared to wild-type (WT) PRLR (Figs. 4A, 5A and Supplementary Material, Fig. S2). Cells expressing WT PRLR had the expected prolactin-induced increases in pSTAT5 (Figs. 4B and 5B) and pAkt expression (Figs 4C and 5C), transcription of CISH (Figs 4D and 5D) and cell proliferation (Figs 4E and 5E). In contrast, the loss-of-function PRLR ECD mutant His188Arg abolished pSTAT5 expression (Figs 4B and 5B) and CISH transcription (Figs 4D and 5D), consistent with previous reports (25,29), and impaired the pAkt response (Figs 4C and 5C), but did not affect proliferation (Figs 4E and 5E).

Cells expressing the prolactinoma-associated PRLR ECD variant Ile76Val (Fig. 4B–E) and the ICD variants Glu376Gln and Arg453Trp (Fig. 5B–E) had similar responses to those of WT cells, indicating that these are likely to be benign polymorphisms (Supplementary Material, Table S1). Cells expressing the Gly57Ser and Ile146Leu PRLR ECD variants showed decreased transcription of CISH (Fig. 4D) and increased pSTAT5 expression (Fig. 5B), respectively (Fig. 4D and F), although these altered responses were only observed at supraphysiological prolactin concentrations, i.e. 500–1000 ng/mL, which are ~17–34 times the upper limit of normal concentrations in females (Fig. 1). In contrast, cells expressing the ICD variant Asn492Ile showed increased pAkt expression (Fig. 5C) and proliferation, as assessed by CellTiter Blue assays (Fig. 5E and Supplementary Material, Table S1), at much lower prolactin concentrations (50–200 ng/mL). This increased proliferation of cells expressing the Asn492Ile PRLR variant was confirmed by measuring BrdU incorporation (Fig. 6A–B). Apoptosis, assessed using a Caspase-Glo-3/7 assay, was not altered in cells expressing the Asn492Ile variant, when compared to those expressing WT PRLR, but was significantly increased in cells expressing the His188Arg mutant PRLR following 96 h of treatment with 200 ng/mL PRL (Fig. 6C). These results indicate that the Asn492Ile PRLR is most likely to be a rare pathogenic variant with a role in the etiology of prolactinomas (Supplementary Material, Table S1).

### Effects of the mTOR inhibitor everolimus on Asn492Ile PRLR functional activity

**Figure 7:**
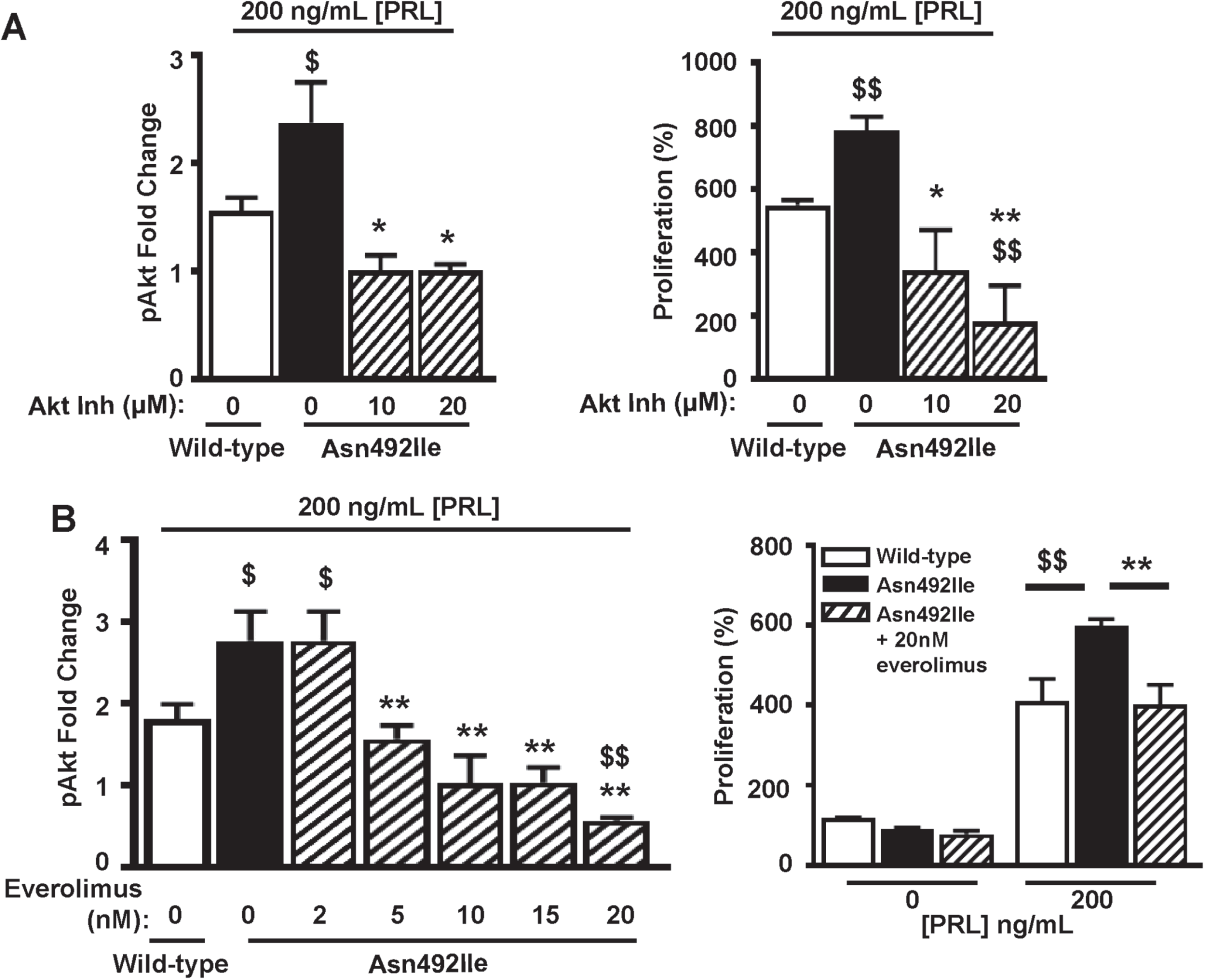
Effects of inhibitors of the Akt signaling pathway on Asn492Ile PRLR-induced proliferation. (**A**) Effect of the Akt1/2 inhibitor (inh) (1,3-Dihydro-1-(1-((4-(6-phenyl-1H-imidazo[4,5-g]quinoxalin-7-yl)phenyl)methyl)-4-piperidinyl)-2H-benzimidazol-2-one trifluoroacetate salt hydrate) on pAkt responses (left panel) and 96 h proliferation (right panel) in HEK293 cells expressing WT and Asn492Ile PRLR and treated with 200 ng/mL PRL. The Akt inhibitor reduced pAkt responses and proliferation in Asn492Ile PRLR-expressing HEK293 cells such that they were not significantly different to WT cells. (**B**) Effect of the mTOR inhibitor, everolimus, on pAkt responses (left panel) and 96 hour proliferation (right panel) in WT and Asn492Ile PRLR-expressing HEK293 cells treated with 200 ng/mL PRL. Everolimus reduced pAkt responses and proliferation in Asn492Ile PRLR-expressing HEK293 cells such that they were not significantly different to responses in WT cells. Untreated WT (open bars) and mutant (filled bars) PRLRs, and inhibitor-treated mutant PRLRs (hatched bars). Mean ± SEM from four biological replicates shown with ^$^*P* < 0.05, ^*^P < 0.05, ^$$^*P* < 0.02 and ^**^P < 0.02 for comparisons to WT (dollar) and comparisons to Asn492Ile (asterisk).

The finding that the prolactinoma-associated Asn492Ile PRLR variant resulted in a gain of function that increased pAkt signaling, which is known to have a role in the etiology of other neoplasms (e.g. carcinomas of the breast, ovary, colon, pancreas and liver, non-Hodgkin’s lymphoma and pituitary tumors; 2,32–37), and proliferation indicated that it may have a role in the development of prolactinomas via prolactin-induced Akt activation. We therefore hypothesized that targeting of this pathway may represent an effective therapy for prolactinoma in patients with this PRLR variant. We assessed the effects of an Akt1/2 inhibitor and the mTOR inhibitor, everolimus, which is a Food and Drug Administration-licensed drug for use in a number of cancers (37) and acts on the Akt pathway (38), on pAkt and proliferation responses in cells expressing the WT PRLR (Supplementary Material, Fig. S3) and mutant Asn492Ile PRLR (Fig. 7). Treatments with an Akt inhibitor or everolimus were found to significantly reduce the PRL-induced increases in pAkt and proliferation by the WT PRLR (Supplementary Material, Fig. S3). Moreover, the prolactin-induced elevations in pAkt activity and proliferation that were associated with the Asn492Ile mutant PRLR could also be reduced to similar levels to those of cells expressing WT PRLR, by a concentration of 10 μm Akt1/2 inhibitor or 20 nM everolimus (Fig. 7). In contrast, everolimus had no effect on the pSTAT5 pathway in WT or mutant Asn492Ile PRLR-expressing cells, thereby demonstrating its specificity for the pAkt pathway (Fig. S4). Thus, these results indicate that PRLR-mediated pAkt signaling, whose physiological significance in the lactotroph is unknown, may have a role in the development of some prolactinomas.

### Serum prolactin concentrations in healthy individuals with ECD and ICD *PRLR* variants

**Figure 8:**
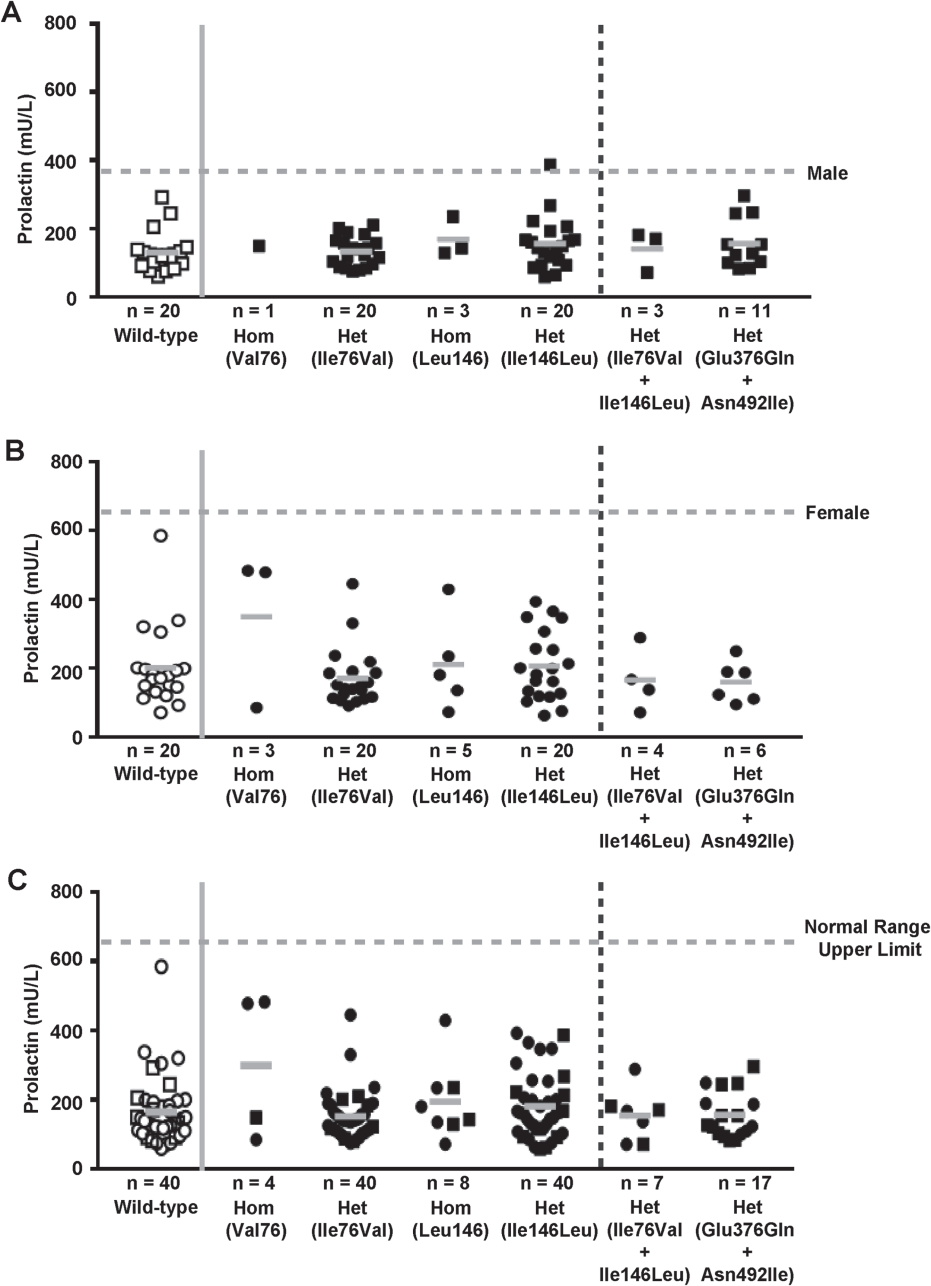
Serum prolactin concentrations of individuals from the OBB data set with *PRLR* variants. (**A**) Males (filled squares). (**B**) Females (filled circles). (**C**) Combined males and females. Males and females, with four of the six PRLR variants from the OBB cohort (Table 1), are grouped by homozygous (hom) and heterozygous (het) genotypes. Individuals having only the WT alleles (i.e. haplotype Ile76/Ile146/Glu376/Asn492) were used as controls (open squares, male; open circles, female) and are shown left of the vertical solid gray line. Prolactin was measured in samples available from 40 (20 males and 20 females) of the >6600 individuals homozygous for the WT alleles; 4 of the 7 individuals homozygous for Val76, and 40 (20 males and 20 females) of the 404 individuals heterozygous for Ile76Val; 8 of the 10 individuals homozygous for the Ile146, and 40 (20 males and 20 females) of the 402 individuals heterozygous for Ile146Leu; 7 of the 8 individuals with the Ile76Val/ Ile146Leu haplotype; and 17 of the 28 individuals with the Glu376Gln/ Asn492Ile haplotype (Table 1). In addition, only one patient had the Gly57Ser PRLR variant, and this was not included in the study. Individual measurements are shown with mean values indicated by a solid gray bar. Prolactin concentrations in individuals with more than one PRLR variant are shown right of the vertical broken line. Upper limit of the prolactin normal ranges for males (45–375 mU/L) and females (60–625 mU/L) is shown by the horizontal broken gray line. For panel C, the upper limit of the prolactin normal range for females is shown. Individuals heterozygous or homozygous for the minor allele of Ile76Val and Ile146Leu, which each had a prevalence of <5% in the OBB cohort, and individuals heterozygous for the co-occurring Glu376Gln and Asn492Ile variants, did not have hyperprolactinaemia.

The enrichment of some germline *PRLR* variants (e.g. Glu376Gln and Asn492Ile) (Table 1) in patients with prolactinomas, together with our previous observation that individuals harboring a loss-of-function mutant Arg188His PRLR developed hyperprolactinemia (25), led us to hypothesize that asymptomatic, healthy (i.e. normal) individuals with such *PRLR* variants may have alterations in serum prolactin concentrations. To investigate this, we utilized the OBB cohort of 7045 healthy individuals (3324 males and 4316 non-pregnant females), who are aged between 30 and 50 years and do not have a cardiovascular disease, an untreated malignancy or an ongoing systemic disease (31). Examination of the available exome chip data revealed the occurrence of four of the six *PRLR* variants identified in the prolactinoma patient cohort (Ile76Val, Ile146Leu, Glu376Gln and Asn492Ile) in >1 individual (Table 1 and Fig. 8). These *PRLR* variants occurred at frequencies similar to those observed in other control cohorts of European descent (e.g. ExAc and 1000 Genomes). Thus, >400 individuals were heterozygous for the *PRLR* ECD variants (Ile76Val, *n* = 404, and Ile146Leu, *n* = 402) and <10 individuals were homozygous for the minor allele (Ile76Val, *n* = 7; Ile146Leu *n* = 10). However, measurement of prolactin from available sera revealed that neither the homozygous nor the heterozygous individuals for these Ile76Val or Ile146Leu *PRLR* variants had significant alterations in serum prolactin concentrations (Fig. 8). Similarly, individuals heterozygous for the rare ICD *PRLR* variants, Glu376Gln (*n* = 17) and Asn492Ile (*n* = 17), did not have significant alterations in serum prolactin concentrations when compared to those without the variant (Fig. 8). These results (Fig. 8) together with the observed effects on pSTAT5 and pAkt signaling (Figs 4 and 5 and Supplementary Material, Table S1) of these variants indicate that the ICD rare variant Glu376Gln and the ECD low-frequency variants Ile76Val and Ile146Leu are likely benign polymorphisms and that the ICD rare variant Asn492Ile may be a likely low penetrance risk allele for the occurrence of prolactinoma.

## Discussion

Our study has (1) identified an association between the occurrence of prolactinoma and two germline PRLR variants, Glu376Gln and Asn492Ile, which are rare and in complete LD (Table 1 and Fig. 1); (2) shown that the Asn492Ile PRLR variant results in a gain of function, with increased signaling by the Akt pathway and proliferation (Fig. 5); and (3) shown that an Akt inhibitor and everolimus, an mTOR inhibitor, can normalize the increased activity associated with the Asn492Ile PRLR variant (Fig. 7), thereby providing a potential new treatment option for patients.

Our findings of an association between PRLR variants and prolactinoma (Table 1) differ from those reported by a French study that did not find an association between *PRLR* variants and prolactinomas in 88 patients (29). Importantly, the French study did not identify any of the three PRLR ICD rare variants (Glu376Gln, Arg453Trp and Asn492Ile) that were found in our study to have the strongest association with prolactinomas (Table 1) (29). This suggests that the two studies may contain different subpopulations, and indeed, our study contains patients with an older median age of onset, more males and possibly a higher number of dopamine-agonist resistant prolactinomas that required surgery, which were not reported in the French study (29). In addition, the two cohorts may have differences in ancestry that may contribute to these variations in allele frequencies. For example, marked differences in rare germline variant frequencies have been observed between disease cohorts in studies of other endocrine tumors such as pheochromocytoma/paraganglioma (PPGL) (39–42). Thus, in these patients, the frequency of succinate dehydrogenase complex, subunit A (SDHA) variants, was reported to differ markedly between PPGL cohorts, with the highest frequency observed in the Dutch population, which may potentially be due to the presence of a founder mutation Arg31Stop in SDHA (39–42).

Our results, which show a role for a PRLR mutation in the development of human prolactinomas (Table 1, Figs 1, 5, 6 and 7), are in agreement with the findings from mouse studies, which have reported that conventional *Prlr* knockout mice develop prolactinomas (26), although there is an apparent paradox in that both gain of function (in humans) and loss of function (in mouse) of PRLR can lead to prolactinoma formation. Possible explanations for this paradox likely involve the different mechanisms for regulating the multiple PRLR signaling pathways (Supplementary Material, Fig. S1). Thus, in the *Prlr* knockout mice, the loss of negative feedback signaling on the lactotroph cell involving PRLR and diminished or absent STAT5 signaling is likely to be driving tumor development, whereas in humans, activation of the mTOR/Akt signaling may represent an alternate tumorigenic mechanism. In addition, the different signaling pathways downstream of PRLR may be activated in a tissue-specific manner and lead to different physiological effects. For example, deletion of *Prlr* in different subtypes of TIDA neurons (i.e. GABAergic versus dopaminergic) of the arcuate nucleus and lactotroph-specific *Prlr* knockout in mice have revealed that the PRLR may have distinct physiological outputs in these different cell types (16,26). Thus, the Akt pathway may mediate PRL-specific responses in one cell type, while having no function or a different function in another cell type and the local expression of different PRLR isoforms may also impact on whether Akt signaling is activated by the PRLR (9).

Analysis of data available from the OBB cohort of healthy individuals revealed that four (Ile76Val, Ile146Leu, Glu376Gln and Asn492Ile) of the six germline PRLR variants identified in the prolactinoma patients (Table 1 and Supplementary Material, Table S1) occurred in >1 individual. However, these PRLR variants were not associated with alterations in serum prolactin concentrations in healthy individuals (Fig. 8). The low-frequency PRLR variants Ile76Val and Ile146Leu, which did not alter PRLR function or only altered PRLR function at supraphysiological prolactin concentrations (Fig. 4), respectively, likely represent PRLR benign polymorphisms, without clinical significance, and this is in agreement with recent reports from studies of women with breast cancer and fibroadenomas (43). The rare Glu376Gln PRLR variant, which did not alter PRLR function (Fig. 5) but is in complete LD with the gain-of-function Asn492Ile mutant PRLR, is also likely to be a benign polymorphism, despite its highly significant association with prolactinomas (Table 1). However, the possibility remains that this and other rare variants may have an effect on other pathways, such as the Ras/Raf MAPK and Src kinase pathways, which have been reported to be involved in PRLR signaling (44). In addition, these PRLR variants may have effects on receptor trafficking and degradation, which were not investigated by our study, and it therefore remains a possibility that these PRLR variants may have subtle effects on prolactinoma development *in vivo* that may not have been detectable by our *in vitro* assays. The absence of elevated prolactin levels in individuals who have the rare Asn492Ile gain-of-function mutant PRLR in the OBB cohort indicates that this allele likely represents a low penetrance risk allele for the occurrence of prolactinoma and that the majority of individuals harboring this variant will remain asymptomatic. This phenomenon has been observed for other genes associated with endocrine tumors (e.g. the SDHA in paraganglioma and cadherin-related 23 in pituitary adenomas) in which rare germline heterozygous coding variants are overrepresented in cases relative to controls but are associated with apparent low-disease penetrance (39,45).

Our studies have highlighted that a gain of functional activity within the pAkt pathway may be an important mechanism in pituitary tumorigenesis in patients with the Asn492Ile PRLR, and this may be analogous to the increased pAkt signaling that has been reported to have an etiological role in other neoplasms (e.g. in carcinomas of the breast, ovary, colon, pancreas and liver, non-Hodgkins Lymphoma and pituitary tumors; (2,32–37)). Furthermore, our results demonstrated that inhibition of this pathway by pAkt or mTOR inhibition can normalize signaling and decrease proliferation of cells expressing the Asn492Ile variant. Thus, our findings indicate that everolimus, an mTOR inhibitor, which has been used for treating patients with rare pituitary carcinomas (46–48), may represent an effective therapy for the >10% of prolactinomas that are resistant to dopamine agonist therapy (49,50). Indeed, our observations that the PRLR Asn492Ile gain-of-function mutation was frequently observed in prolactinoma patients requiring pituitary surgery suggest a potential personalized treatment approach for patients whose prolactinomas are aggressive or do not respond to dopamine-agonist therapy. Thus, such patients could be offered *PRLR* genotyping and if a germline gain-of-function mutation (e.g. Asn492Ile) is found, then it may be appropriate to offer such patients medical treatment with everolimus rather than proceeding directly to surgery.

In summary, our studies have identified that a gain-of-function PRLR mutation, which activates pAkt signaling, is associated with prolactinomas and that everolimus may represent a potential effective treatment in patients with prolactinomas resistant to currently used medical treatments.

## Materials and Methods

### Patients and mutational analysis

Informed consent for DNA sequence analysis was obtained from all patients with the use of protocols approved by local and national research ethics committees (MREC/02/2/93). DNA was extracted from blood and tumor samples using the DNeasy Blood and Tissue Kit (Qiagen) according to the manufacturer’s protocol. Polymerase chain reaction (PCR) amplification of the coding region, untranslated regions and intron–exon boundaries (i.e. ~30 bp 5′ and ~30 bp 3′ of each exon) of the *PRLR* gene was performed using gene-specific primers (SigmaAldrich). Dideoxynucleotide sequencing using the BigDye Terminator v3.1 Cycle Sequencing Kit (Life Technologies) and an automated detection system (ABI 3730 Automated capillary sequencer; Applied Biosystems) was performed, as previously described (25). The population frequencies of the PRLR single nucleotide variants (SNVs) were evaluated using the ExAc data set (http://exac.broadinstitute.org/) (51). ExAc contains DNA sequence data from 60 706 normal individuals (August 2017) and patients with disorders that include diabetes mellitus type 2, heart disease, inflammatory bowel disease and endocrine cancers (e.g. adrenocortical carcinoma, papillary thyroid carcinoma, paraganglioma and pheochromocytomas 51), but not pituitary tumors. SIFT (http://sift.jcvi.org/) and Polyphen-2 (http://genetics.bwh.harvard.edu/pph2/) were used to predict the effect of amino acid substitutions (52,53). Amino acid conservation was examined in PRLR orthologs using *ClustalW2* (www.ebi.ac.uk/Tools/msa/clustalw2/) (54). Comparisons between the frequency of variants in the ExAc cohort and the prolactinoma cohort were performed by Fisher’s exact test, and Bonferroni correction performed for multiple testing, using GraphPad Prism. Data regarding common and low-frequency/rare variants were derived from the ExAc cohort, which comprises 60 706 individuals (51), and the OBB cohort (31), which comprises 7045 individuals (see below).

### OBB cohort

The OBB cohort (http://www.oxfordbiobank.org.uk) consists of an age-stratified random sample of men and women (aged 30–50 years) from Oxfordshire, UK. All participants were of white, European origin and did not have a cardiovascular disease, an untreated malignancy or a current systemic disease (31). No participants were pregnant at the time of sample collection. Data collection has been described previously (31). All participants gave written, informed consent to participate, and studies were approved by the Oxfordshire Research Ethics Committee. Data and DNA were available on 7045 individuals for this study. Genotyping was performed using the Illumina HumanExome Beadchip. Prolactin measurements were performed on available serum samples using an assay (ADVIA Centaur System, Bayer). This assay, which has minimal pick-up of macroprolactin, has reference ranges of 45–375 mU/L for males and 60–625 mU/L for females with an intra-run coefficient of variation = 2.3–3.3% and inter-run coefficient of variation = 1.4–4.7%.

Mean prolactin values from individuals with each of the variants were compared with values from 40 individuals (20 males and 20 females) with the WT alleles only (i.e. controls), by Student’s *t*-test. Comparisons between the frequency of variants in the prolactinoma cohort and the OBB cohorts were performed using Fisher’s exact test and Bonferroni correction for multiple comparisons.

### Plasmids, antibodies and cell culture

The full-length sequence-verified pd*EYFP-PRLR* WT construct was obtained from Source Bioscience. Each mutant *PRLR* construct was generated using the Quikchange Lightning Site-directed Mutagenesis kit (Agilent Technologies) and sequence-specific primers (SigmaAldrich) (25). The pGL4.10-*CISH* promoter vector, described previously, was used in luciferase reporter assays (25).

For immunofluorescence and western blot analysis, the following antibodies were used: primary antibodies rabbit anti-PRLR (H-300, 1:1000, SantaCruz Technologies), anti-BrdU (1:200, Abcam) and rabbit anti-alpha-tubulin (1:1000, Abcam); secondary antibodies Alexa Fluor 488 (1:500, Molecular Probes) and anti-rat Cy3 (1:300, Molecular Probes) for immunofluorescence studies; and Horseradish peroxidase (HRP)-conjugated goat-anti-rabbit (1:3000, Biorad) for western blot analysis. HEK293 cells were grown in 10% fetal calf serum (FCS)-treated DMEM-Glutamax medium (Gibco) and maintained at 37°C, 5% CO_2_. Functional studies were carried out on poly-L-lysine treated cells, and transient transfections used Lipofectamine 2000 (Invitrogen).

### Confocal imaging

Confocal imaging was performed as previously described (25). Cells were plated in 6 well plates and transfected with 1000 ng WT or mutant PRLR expression constructs. Cells were fixed in 4% paraformaldehyde/PBS (SigmaAldrich), permeabilized with 1% TritonX-100/PBS (Thermo Scientific) and blocked in donkey serum, followed by immunostaining with anti-PRLR and Alexa Fluor 488. Cells were mounted in Vectashield plus 4,6-diamidino-2-phenylindole **(**DAPI) (Vector Labs) that stains nuclei. Images were captured using a confocal, two-photon laser-scanning microscope (Zeiss, LSM 510 META) with a Plan-Achromat ×63/1.4 oil DIC objective (25). An argon laser (488 nm) and 740 nm two-photon laser were used to excite Alexa Fluor 488 and DAPI fluorescence, respectively (25). Emission of Alexa Fluor 488 and DAPI was detected within a spectral detection range of 509–550 nm and 415–501 nm, respectively.

### AlphaScreen surefire assays

AlphaScreen assays were performed as previously described (25). Cells were transiently transfected in 48 well plates with 200 ng of either WT or mutant PRLR vectors. After 30 h, cells were incubated in serum-free media for 12 h prior to treatment with human recombinant prolactin for 20 min at concentrations ranging from 0 to 1000 ng/mL. For AlphaScreen studies with the Akt1/2 inhibitor (A6730, Sigma), cells were pre-treated with DMSO vehicle or Akt1/2 inhibitor for 30 min (55). For studies with everolimus, cells were treated with DMSO vehicle or everolimus when prolactin was added to the cells. In all experiments, cells were lysed in 1× Surefire lysis buffer and AlphaScreen Surefire pSTAT5 or pAkt assays performed according to manufacturer’s instructions (56). The fluorescence signal was measured using the PHERAstar *FS* microplate reader (BMG Labtech). A minimum of four independent biological replicates were used for each condition within each experiment. Data were plotted as fold-change responses relative to the response at 0 ng/mL in cells expressing the WT PRLR expression construct, and statistical analyses performed using two-way analysis of variance (ANOVA) with Tukey’s multiple-comparisons test. The variability between fold-change responses for the same PRLR plasmid can be wide on different days due to temperature changes and batch variability between kits. Therefore, each vector was only compared to others analyzed on the same day, using a single kit, and measured on the same plate to minimize and control variability.

### Luciferase reporter assays

Luciferase reporter assays were performed as previously described (25). HEK293 cells were transiently co-transfected in 48 well plates with 50 ng of pGL4.10-CISH reporter gene construct, 10 ng of pRL (renilla) control vector and 100 ng of WT or mutant PRLR vectors. Following transfection, cells were incubated in serum-free media overnight. Cells were then treated with 0–500 ng/mL prolactin for 24 h in serum-free media. Cells were lysed and assayed for luciferase activity using a Turner Biosystems luminometer and the Dual-Luciferase Reporter assay system (Promega). The firefly luciferase activity was adjusted for Renilla luciferase activity (Firefly/Renilla ratio) and ratios expressed as a fold-change relative to cells treated with 0 ng/mL prolactin within each group. Expression of PRLR in the transfected cells was confirmed by western blot analysis. A minimum of four independent biological replicates were performed in each experiment. Statistical analysis was performed by two-way ANOVA with Tukey’s multiple-comparisons test comparing responses to prolactin in each group to that of WT expressing cells.

### Western blot analysis

Western blot analysis was used to assess expression of transfected PRLR and endogenous α-tubulin as a loading control in lysates from AlphaScreen and luciferase reporter assays. Lysates were resuspended in Laemmli buffer and boiled and separated on 10% sodium-dodecyl sulfate polyacrylamide gel electrophoresis gels. Following transfer to polyvinylidene difluoride membrane (Amersham), blots were blocked in 5% marvel/TBS-T, then probed with anti-PRLR (SantaCruz) and anti-α-tubulin (Abcam) antibodies. Blots were visualized using the Immuno-Star WesternC kit (BioRad) on a BioRad Chemidoc XRS+ system (25).

### Proliferation assays

HEK293 cells were plated in 96 well plates and transfected with 50 ng WT or variant PRLR vectors per well. Following 24 h, cells were treated with 200 ng/mL prolactin and proliferation assessed every 24 h for 96 h using the CellTiter Blue kit (Promega) (57). The cell count for day 1 (i.e. time 0 before prolactin was added) was set as 100%. Prolactin was then added to cells, and a cell count was taken every 24 h until 96 h had elapsed. Each cell count was expressed relative to the original cell count, which was before the addition of prolactin. Plates were read on a PHERAstar *FS* microplate reader (BMG Labtech). For studies with the Akt1/2 inhibitor or everolimus, drugs were added to the cells at the same time as prolactin. Proliferation was also assessed by BrdU incorporation, as follows. HEK293 cells were plated in 6 well plates with coverslips and transfected with 50 ng WT or variant PRLR vectors per well. Following 24 h, cells were treated with 200 ng/mL prolactin and incubated, and at 96 h, the cells were then exposed to BrdU for 15 min before fixation in 4% paraformaldehyde/ PBS. Cells were permeabilized with 1% TritonX-100/PBS (Thermo Scientific) and blocked in donkey serum, followed by immunostaining with anti-BrdU primary antibody and anti-rat Cy3 secondary antibody. Cells were mounted in Prolong Gold Antifade reagent with DAPI (Invitrogen). Imaging was performed using an Eclipse E400 fluorescence microscope, and images were captured using a DXM1200C digital camera and NIS Elements software (Nikon). The number of BrdU-positive cells was quantified using ImageJ (NIH). Statistical analysis was performed by one-way ANOVA.

### Apoptosis assay

HEK293 cells were plated in 96 well plates and transfected with 50 ng WT or variant PRLR vectors per well. Following 24 h, cells were treated with 0 ng/mL or 200 ng/mL prolactin and apoptosis was assessed at 0 h and 96 h using the Caspase-Glo-3/7 kit (Promega) (57). Statistical analysis was performed by one-way ANOVA.

### Statistics

Comparisons between the frequency of variants in the prolactinoma cohort and the ExAC and OBB cohorts were performed using Fisher’s exact test. Mean prolactin values from patients with each of the PRLR variants were compared with values from patients with the WT alleles by Student’s *t*-test. A minimum of four independent biological replicates were performed in all cell-based assays. Statistical analyses were performed by two-way ANOVA with Tukey’s multiple-comparisons test for AlphaScreen and luciferase reporter assays and by one-way ANOVA for BrdU and apoptosis assays.

## Supplementary Material

Supplementary Data
