# Supplementary material for: Association of prolactin receptor (PRLR) variants with prolactinomas
Source: Hum Mol Genet. 2018 Nov 15;28(6):1023–37. doi: 10.1093/hmg/ddy396 (PMC6400049; doi:10.1093/hmg/ddy396)
Supplement: Supplementary Data [file ddy396_clean_supplementary_material.pdf]

SUPPLEMENTARY INFORMATION

Association of Prolactin Receptor (*PRLR*) Variants with Prolactinomas

Caroline M. Gorvin, Paul J. Newey, Angela Rogers, Victoria Stokes, Matt J. Neville, Kate E. Lines, Georgia Ntali, Peter Lees, Patrick J. Morrison, Panagiotis N. Singhellakis, Fotini Ch Malandrinou, Niki Karavitaki, Ashley B. Grossman, Fredrik Karpe, Rajesh V. Thakker

Formatted: Not Highlight

SUPPLEMENTARY FIGURES

Fig. S1: Schematic diagram of the full-length prolactin receptor (PRLR) showing the location of variants examined in these studies

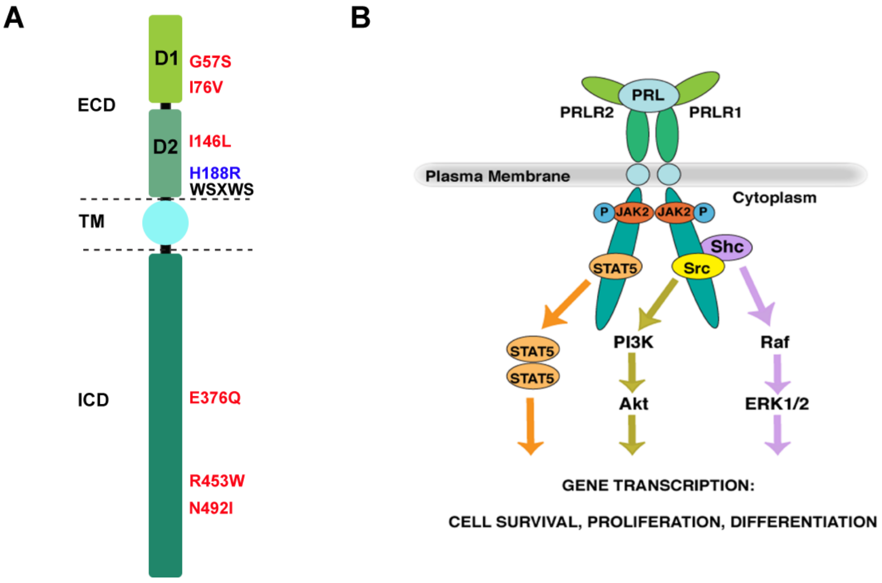

(A) Schematic diagram of PRLR showing the major domains and location of PRLR variants investigated in this study (red - identified in prolactinoma patients (Table 1) and blue - previously reported in familial hyperprolactinaemia (1)). The extracellular domain (ECD) (residues 1-210) can be divided into subdomains designated D1 (residues 1-101) and D2 (residues 109-210). D2 contains the WSXWS motif (residues 191-195) that is conserved in cytokine receptors (2-4). The transmembrane domain (TM) comprises residues 211-234 and the intracellular domain (ICD) consists of residues 235-598. Dashed lines indicate plasma membrane. Residue numbers are from PRLR without the signal peptide. The 7 PRLR variants investigated in this study consisted of 4 ECD variants and 3 ICD variants. The 4 ECD PRLR variants comprised: Gly57Ser (G57S), located in D1; Ile76Val (I76V), located in the D1 domain, previously reported as a gain-of-function variant (5); Ile146Leu (I146L), located in the D2 domain, also previously reported as a gain-of-function variant (5); and His188Arg (H188R) (blue) located in proximity to the high-affinity ligand-binding domain

Field Code Changed

Field Code Changed

Field Code Changed

Field Code Changed

and previously demonstrated to be a loss-of-function variant associated with hyperprolactinaemia (1). The 3 PRLR ICD variants, which were located distal to the Janus kinase 2 (JAK2) binding site (residues 243-250) (6, 7), comprised Glu376Gln (E376Q), Arg453Trp (R453W) and Asn492Ile (N492I). (B) Schematic diagram of PRLR signaling pathways. Prolactin binding has been reported to induce PRLR activation and JAK2 phosphorylation that activates three pathways: signal transducer and activator of transcription 5 (STAT5), phosphatidylinositol-3-kinase (PI3K)/ protein kinase B (PKB), a serine/ threonine kinase referred to as Akt, and extracellular signal-regulated kinase 1 and 2 (ERK1/2) that modify gene transcription to effect cell survival, proliferation and differentiation. Although these pathways are predominantly activated by the full-length PRLR isoform, a number of shorter PRLR isoforms may be generated by alternative splicing, and may influence receptor activity either through sequestering ligand or through heterodimerization with the full-length PRLR (6) pERK1/2 signaling was not investigated in this study, as our previous studies have demonstrated the PRLR does not signal via this pathway in transfected HEK293 cells (1).

Field Code Changed

Field Code Changed

Field Code Changed

Field Code Changed

Fig. S2: Western blot analysis of lysates from AlphaScreen, CISH luciferase reporter and proliferation assays of PRLR variants

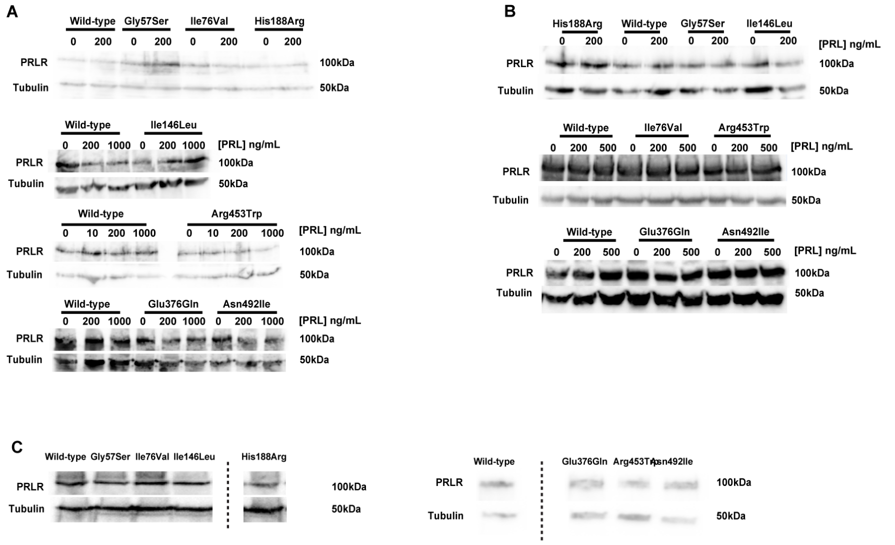

(A) AlphaScreen assays, (B) CISH luciferase reporter assays, (C) Proliferation assays, with dotted lines indicating where blots have been cropped. Representative lysates show approximately equal expression levels of PRLR in each transfected cell type. Tubulin was used as a loading control.

Figure S3: Effects of the Akt inhibitor and everolimus on PRL-induced increases in pAkt signalling and proliferation in PRLR WT expressing cells

Formatted: Not Highlight

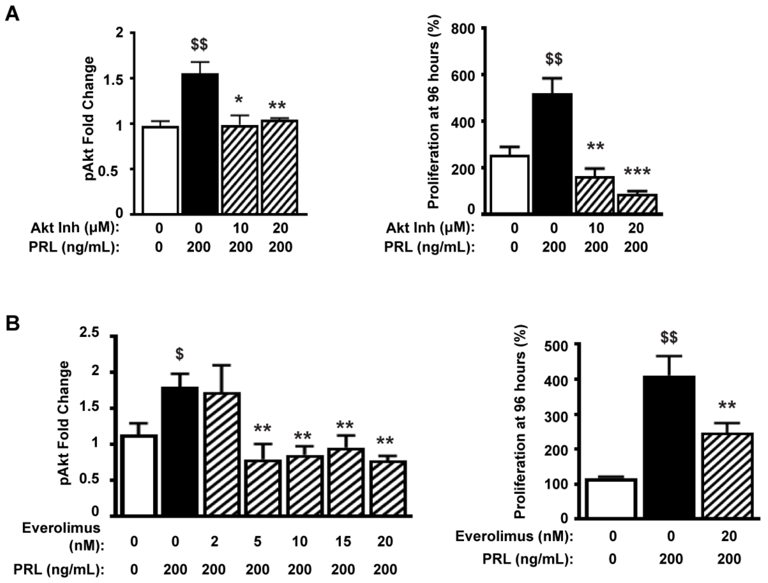

(A) Effect of the Akt1/2 inhibitor (inh) (1,3-Dihydro-1-(1-((4-(6-phenyl-1H-imidazo[4,5-g]quinoxalin-7-yl)phenyl)methyl)-4-piperidinyl)-2H-benzimidazol-2-one trifluoroacetate salt hydrate) on pAkt responses (left panel) and 96 hour proliferation (right panel) in HEK293 cells expressing WT PRLR and treated with 0 ng/mL or 200 ng/mL prolactin (PRL). The Akt inhibitor reduced pAkt responses and proliferation in WT PRLR expressing HEK293 cells such that they were not significantly different to cells treated with 0 ng/mL PRL. (B) Effect of the mammalian target of rapamycin (mTOR) inhibitor, everolimus, on pAkt responses (left panel) and 96 hour proliferation (right panel) in WT PRLR expressing HEK293 cells treated with 0 ng/mL or 200 ng/mL PRL. Everolimus reduced pAkt responses and proliferation in WT PRLR expressing cells. PRLR WT cells treated with 0 ng/mL PRL (open bars), 200 ng/mL PRL (filled bars), and inhibitor-treated (hatched bars). Mean±SEM from 4 biological replicates shown with $^{\$}p<0.05$, $*p<0.05$, $^{\$\$}p<0.02$ and $**p<0.02$ for comparisons to 0 ng/mL (dollar) and comparisons to 200 ng/mL PRL (asterix).

Formatted: Not Highlight

Fig. S4: Effect of everolimus on pSTAT5 signaling

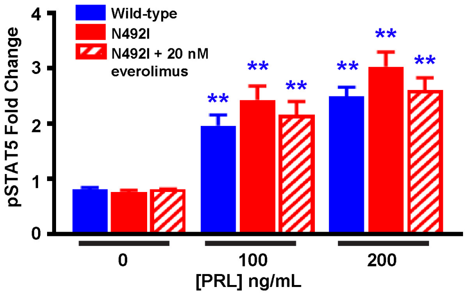

Effect of the mTOR inhibitor everolimus on pSTAT5 responses in wild-type and Asn492Ile PRLR cells treated with prolactin. Everolimus had no effect on pSTAT5 responses and Asn492Ile PRLR responses were not significantly different to wild-type responses. Statistical significance comparing responses to basal levels in each of the groups are shown. **p<0.02. There was no significant difference between wild-type and Asn492Ile responses. **p<0.02. N=4 biological replicates for each point. Error bars indicate SEM.

Table S1: Summary of effects of PRLR variants identified in prolactinoma patients on *in vitro* functions of pSTAT5 and pAkt signaling, transcription of a CISH reporter gene, and cellular proliferation.

	PRLR Variant[a]	Cell Localisation[b]	Immediate Effects[b] (AlphaScreen)		Late Effects (Luciferase Reporter)[b]	
			pSTAT5	pAkt	CISH	Proliferation
	WT	++	++	++	++	++
ECD	Gly57Ser	++	++	++	+	++
ECD	Ile76Val	++	++	++	++	++
ECD	Ile146Leu	++	+++	++	++	++
ECD	His188Arg	++	-	+	-	++
ICD	Glu376Gln	++	++	++	++	++
ICD	Arg453Trp	++	++	++	++	++
ICD	Asn492Ile	++	++	+++	++	+++

[a]The ECD and ICD locations of 6 PRLR variants (Gly57Ser, Ile76Val, Ile146Leu, Glu376Gln, Arg453Trp and Asn492Ile) found in prolactinoma patients and a control loss-of-function mutant (His188Arg), are shown. WT - Wild-type. [b] +++, gain-of-function; ++, normal; +, impaired; -, abolished. The specific PRLR signaling responses of each variant are as follows. Of the ECD variants: the Gly57Ser rare variant impaired CISH reporter activity only; the Ile76Val and Ile146Leu low-frequency variants, previously reported to be constitutive gain-of-function mutations that increased pSTAT5 and proliferation (5, 8), respectively, had signaling responses similar to those for WT PRLR or increased pSTAT5 expression only and the reported loss-of-function His188Arg (1) abolished pSTAT5 expression and CISH transcription, and decreased pAkt expression (Figures 1-2) (5, 8). Of the PRLR ICD variants, the novel Arg453Trp variant and the Glu376Gln rare variant were found to have similar early and late signaling responses to those observed for WT PRLR; whereas the Asn492Ile rare variant was associated with significant increases in pAkt expression and proliferation (Figure 2). The absence of any signaling alterations due to the prolactinoma-associated Glu376Gln and Arg453Trp PRLR rare variants may be due to the inheritance of the Glu376Gln variant in LD with the functional Asn492Ile variant, and a lack of a direct involvement of the Arg453Trp variant in tumorigenesis.

Field Code Changed
Field Code Changed
Field Code Changed
Field Code Changed
Field Code Changed
Field Code Changed
Field Code Changed
Field Code Changed

References

1 Newey, P.J., Gorvin, C.M., Cleland, S.J., Willberg, C.B., Bridge, M., Azharuddin, M., Drummond, R.S., van der Merwe, P.A., Klenerman, P., Bountra, C. *et al.* (2013) Mutant prolactin receptor and familial hyperprolactinemia. *N. Engl. J. Med.*, **369**, 2012-2020.

2 van Agthoven, J., Zhang, C., Tallet, E., Raynal, B., Hoos, S., Baron, B., England, P., Goffin, V. and Broutin, I. (2010) Structural characterization of the stem-stem dimerization interface between prolactin receptor chains complexed with the natural hormone. *J. Mol. Biol.*, **404**, 112-126.

3 Broutin, I., Jomain, J.B., Tallet, E., van Agthoven, J., Raynal, B., Hoos, S., Kragelund, B.B., Kelly, P.A., Ducruix, A., England, P. *et al.* (2010) Crystal structure of an affinity-matured prolactin complexed to its dimerized receptor reveals the topology of hormone binding site 2. *J. Biol. Chem.*, **285**, 8422-8433.

4 Svensson, L.A., Bondensgaard, K., Norskov-Lauritsen, L., Christensen, L., Becker, P., Andersen, M.D., Maltesen, M.J., Rand, K.D. and Breinholt, J. (2008) Crystal structure of a prolactin receptor antagonist bound to the extracellular domain of the prolactin receptor. *J. Biol. Chem.*, **283**, 19085-19094.

5 Bogorad, R.L., Courtillot, C., Mestayer, C., Bernichtein, S., Harutyunyan, L., Jomain, J.B., Bachelot, A., Kuttenn, F., Kelly, P.A., Goffin, V. *et al.* (2008) Identification of a gain-of-function mutation of the prolactin receptor in women with benign breast tumors. *Proc. Natl. Acad. Sci. USA*, **105**, 14533-14538.

6 Brooks, C.L. (2012) Molecular mechanisms of prolactin and its receptor. *Endocr Rev*, **33**, 504-525.

7 Lebrun, J.J., Ali, S., Goffin, V., Ullrich, A. and Kelly, P.A. (1995) A single phosphotyrosine residue of the prolactin receptor is responsible for activation of gene transcription. *Proc. Natl. Acad. Sci. USA*, **92**, 4031-4035.

8 Courtillot, C., Chakhtoura, Z., Bogorad, R., Genestie, C., Bernichtein, S., Badachi, Y., Janaud, G., Akakpo, J.P., Bachelot, A., Kuttenn, F. *et al.* (2010) Characterization of two constitutively active prolactin receptor variants in a cohort of 95 women with multiple breast fibroadenomas. *J. Clin. Endocrinol. Metab.*, **95**, 271-279.
